# Ethnomycological study of wild edible and medicinal mushrooms in district Jammu, J&K (UT), India

**DOI:** 10.1186/s13002-022-00521-z

**Published:** 2022-03-24

**Authors:** Roshi Sharma, Yash Pal Sharma, Sayed Azhar Jawad Hashmi, Sanjeev Kumar, Rajesh Kumar Manhas

**Affiliations:** 1 Department of Botany, University of Jammu, Jammu, J&K 180006 India; 2 Department of Botany, Govt. Degree College, Basohli, J&K 184201 India

**Keywords:** Ethnomycological, Cultural importance, Traditional knowledge, Wild edible mushrooms, Jammu

## Abstract

**Background:**

Union Territory of Jammu and Kashmir (J&K) has a rich tradition of usage of wild edible mushrooms (WEMs) for culinary and medicinal purposes. But very few studies, restricted to some regions of the Union Territory, have been conducted to enlist the WEM. District Jammu has never been explored for WEM. Moreover, the quantification of the traditional knowledge of WEM has not been carried out as yet in J&K. Therefore, the present study was conducted in the Jammu district with the aims of enlisting the WEM and its usage, finding the most used WEM, and enumerating the consensus of usage for a species and associated knowledge.

**Methods:**

A total of 192 informants between the age of 25 and 87 years were interviewed. The collected information was organized and arranged based on use reports for quantitative analysis. The cultural importance index (CI) and factor informant consensus (*F*_ic_) were calculated to estimate the cultural importance and to test homogeneity of information and knowledge sharing about WEM, respectively. Analysis of variance was used to evaluate the significance of differences in the usage of WEM among different informant categories.

**Results:**

Results of the present study show that the locals were having knowledge of fourteen fleshy fungi that are mainly utilized for culinary purposes. They also stated various medicinal values of some of these fungi. Agaricaceae and Lyophyllaceae were the most used families, and *Termitomyces* (5 species) was the most represented genus. Based on CI values, *Termitomyces* sp. (CI 0.57) was the most important and diversely used species. *Termitomyces heimii*, *Termitomyces clypeatus*, and *Termitomyces striatus* var. *annulatus* were the other culturally important and frequently consumed species by the locals. More than 78.6% of these WEM were new records as culinary and medicinal for J&K (UT). The values of factor informant consensus (*F*_ic_) varied between 0.98 (culinary) and 0.76 (skin diseases). Females, elders, and informants who have not attended schools were having significantly (*P* < 0.05) higher information regarding WEM.

**Conclusion:**

The inhabitants of district Jammu had good knowledge of WEM, but no documentation, lying of most of the information with elders and uneducated people, and destruction of forests and other natural habitats of WEM pose a serious threat of losing this valuable information in near future. An ardent need is to educate locals regarding regionally available WEM. Further studies are recommended for developing protocols of cultivation of these WEM so that their future availability is ascertained along with creating income resources for the local population.

## Introduction

The edible fleshy fungus growing in natural habitats and not cultivated is classified as wild edible mushroom (WEM). These species are a great source of proteins, fibres, minerals, and trace elements [1] apart from having low content of fats, low or negligible calories and cholesterol [2]. In addition to nutritional values, WEM has abundance of bioactive compounds [3–5]. Due to these nutritional and health benefits, WEM can be used as an important food to eradicate the menace of malnutrition from various African and Asian countries. FAO is also promoting the use of WEM for income generation and food security [6].

As many as 2189 species of edible fungi have been reported to be in use worldwide [7, 8]. A total of 283 edible fungi have been recorded from India [9], besides 100 medicinal fungi [10]. Despite many benefits, the use of WEM is not common in Indian societies due to: (1) incidences of food poisoning after the consumption of toxic fungi, (2) some religious bindings as WEM are considered non-vegetarian food by some communities, (3) urbanization and change in land use from forests to agriculture reducing the availability of WEM, and (4) non-availability of local guide for the identification of edible and toxic fungi. The problem of identification of edible fungus can be solved by promoting the folk taxonomy of the WEM. Folk taxonomies are the outcome of social knowledge, interactions and dialects. It is the categorization of organisms on the basis of the conventional system of using vernacular names [11–13]. Mostly the vernacular names are based on some prominent features such as appearance, colour, habit, habitat, shape, size, smell, taste, and utility as edible or poisonous [11, 12].

Ethnomycological studies on wild edible and medicinal mushrooms have been carried out in different parts of India [9, 10, 14–26], and the world, especially Africa [27–31], but such studies are rare in Jammu and Kashmir [2, 11–13, 32–35]. Quantitative analysis of traditional knowledge using cultural importance index [36] and factor informant consensus [37] has become increasingly popular in recent times. Basically, these analyses show the extent of consensus among an ethnic community for a particular species or knowledge, and the most used species. The present quantitative ethnomycological study is the first of its kind from Jammu and Kashmir.

Jammu is the winter capital of Jammu and Kashmir (Union Territory). The total population of the district is 15,29,958 and a sex ratio of 880 (2011 Census). Most of the inhabitants follow Hindu religion (84.3%) and speak *Dogri* language (70.9%). Ethnically, they are known as *Dogras*. The topography of most part of the district is undulating. Agriculture is the main occupation of approximately 60% of the population. The percentage of uncultivated and cultivated land area is 22.4% and 35.3%, respectively, and the forest cover of district Jammu is merely 12.6% [38]. The forests are highly degraded and fragmented, and the villagers usually visit them for the collection of fuelwood and non-wood forest products especially WEM. The primary aim of the present study is to record the traditional knowledge of wild edible mushrooms of Jammu district. The collected data were utilized to assess the most important WEM used by the local populace and analyse the differences in usage and collection of these WEM among genders, age groups, and education level of informants.

## Material and methods

### Study area

**Fig. 1:**
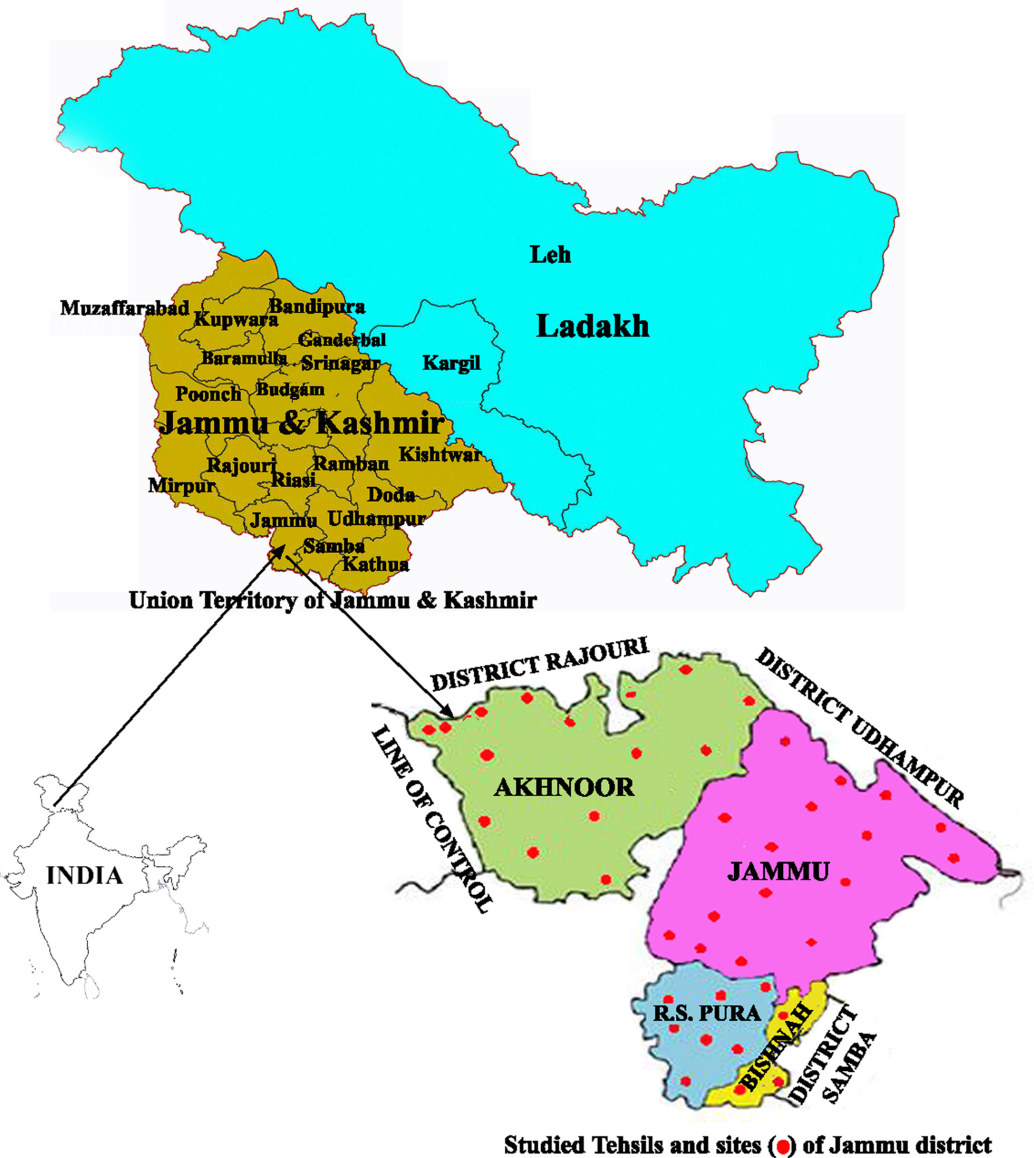
Location map of the study area and sampling points (  )

Jammu is situated to the South of the great Himalayan range and North of the plains of Punjab (Fig. 1). Located at 32.73° N and 74.87° E and covering approximately 3250 km^2^ area, it comprises four tehsils, viz. Akhnoor, Bishnah, Jammu, and Ranbir Singh Pura (R.S. Pura). Altitude of the district above sea level varies from 300 to 800 m. The region has great variation in its temperature and precipitation with mean monthly temperature above 20 °C. Situated in the subtropical part, the district has a markedly periodic climate as is characterized by a dry and increasingly hot season from April to June, a warm monsoon period from July and September and a dry and cold weather from October to December with slight winter rain during the months of January to March. The overall characteristics of Jammu forests is of dry, mixed deciduous or scrub type and the dominant vegetation of the forests comprises of *Acacia modesta*, *Aegle marmelos*, *Butea monosperma*, *Cassia fistula*, *Ziziphus mauritiana*, *Mallotus philippensis*, *Diospyros montana*, *Grewia optiva*, *Pinus roxburghii*, *Premna barbata*, *Terminalia billirica*, *Adhatoda vasica*, *Flacourtia indica*, *Dodonaea viscosa*, *Capparis sepiaria*, *Woodfordia fruticosa*.

### Methodology

#### Collection and identification of fungi

Systematic and periodic surveys of different locations of district Jammu were conducted, and careful field records were made for habitats, hosts, substrates, and photographs of collection sites and fruit bodies were taken for studying wild fleshy fungi. Macroscopic features were studied from fresh material, and microscopic structures were observed in dried material by using 5% potassium hydroxide (KOH) and Congo Red. Microcharacters were observed with a Nikon E-400 microphotographic unit. Further identification and confirmation were done using pertinent keys, monographs and books [39–42]. Details of various mushroom species were taken from Ainsworth and Bisby’s 'Dictionary of Fungi' by Hawksworth et al. [43] and Kirk et al. [44]. Online websites like www.mycokey.com, www.mushroomexpert.com were also used for identification and related information. All the specimens were submitted to the herbarium of the Department of Botany, University of Jammu, Jammu, J&K, India.

#### Ethnomycological data collection

The ethnomycological study was carried out between February 2014 and October 2018, and September 2020 and September 2021. Data were collected from a total of 192 informants (87 females and 105 males) as per semi-structured interviews. All informants were interviewed at least thrice for the collection of information regarding historical background, edibility status, traditional usage, methods of preservation, commercial importance of fleshy fungi, and possible reasons for lower diversity of wild edible fungus in the region. All the interviews and discussions were conducted in different local dialects (*Dogri*, *Hindi, Punjabi*, and *Poonchi*). The verification of the macrofungal species was done in the months of the rainy season, and the informants were requested to escort us during the field visit to confirm the species and information thereof. The help of identified specimens and photographs already with us were also taken.

#### Data analysis

The data, collected through interviews, on the number of uses cited by the informants were analysed using Cultural importance index (CI) and factor informant consensus (*F*_ic_). Cultural importance index (CI) was calculated as the sum total of use report (UR) for a species in culinary and medicinal use categories divided by number (192) of informants (*N*) and mathematically expressed as:

$${\text{CI}} = \sum\limits_{{u = u_{i} }}^{{u_{NC} }} {\sum\limits_{{i = i_{1} }}^{{i_{N} }} {UR_{ui} } } /N$$

where the seven use categories (*u*) are *u*_1&2_ and informants (*i*) are *i*_1*-*192_. According to Tardio and Pardo-de-Santayana [36], CI accounts for the spread as well as versatility of uses. They further stated that CI is a better index than other indices because the maximum value of CI is the total number of uses in different use-categories.

To test homogeneity of information and knowledge sharing about the medicinal plants, the factor informant consensus (*F*_ic_) was used [37]. The *F*_ic_ was calculated as:

$$F_{{{\text{ic}}}} = \frac{{n_{{{\text{ur}}}} - n_{{\text{t}}} }}{{n_{{{\text{ur}}}} - 1}}$$

where *n*_ur_ refers to the total number of citations for a particular use category and *n*_t_ refers to the number of plants used for a particular use category. *F*_ic_ values are low (near 0) if there is no exchange of information about their use among informants, and approach one (1) if information is shared among informants [45–47].

Analysis of variance (ANOVA) was applied to compare the means of different attributes related to informants like gender, age, and education with respect to the collection of WEM and traditional beliefs. The data were normalized using log transformation. Fisher’s least significant difference (LSD) was applied as a multiple range test to compare the significant number of WEM collected by informants when the value of ANOVA was significant at *P* < 0.05.

## Results

A total of 192 informants, 45% females and 55% males, provided information about the wild edible mushrooms (WEMs) of Jammu district. Most of these informants were above the age of 50 years (52%) and literate (68%). Female (4.5, WEM), elderly (4.4, WEM), and illiterate (5.2, WEM) informants reported significantly (*P* < 0.001) higher number of WEM (Table 1).

**Table 1:** The collection of wild edible mushrooms (WEMs) by informants

Attributes	Informants	No. of informants	No. of WEM	ANOVA	
				*F* value	*P* value
Gender	Female	87	4.5^a^ ± 3.2	29.67	< 0.001
	Male	105	3.3^b^ ± 2.3		
Age	Up to 50 years	93	3.3^b^ ± 2.0	24.87	< 0.001
	> 50 years	99	4.4^a^ ± 3.5		
Education	Illiterate	61	5.2^a^ ± 1.5	32.14	< 0.001
	1–8	79	3.4^b^ ± 1.2		
	9–12	42	3.0^c^ ± 1.4		
	> 12	10	2.9^c^ ± 1.4		

The values of WEM given in the table are mean ± SD. Fisher’s least significant difference (LSD) was applied as multiple-range test when analysis of variance (ANOVA) was found significant at *P* < 0.05. Similar alphabets in a column for an attribute show that the values do not vary significantly

**Fig. 2:**
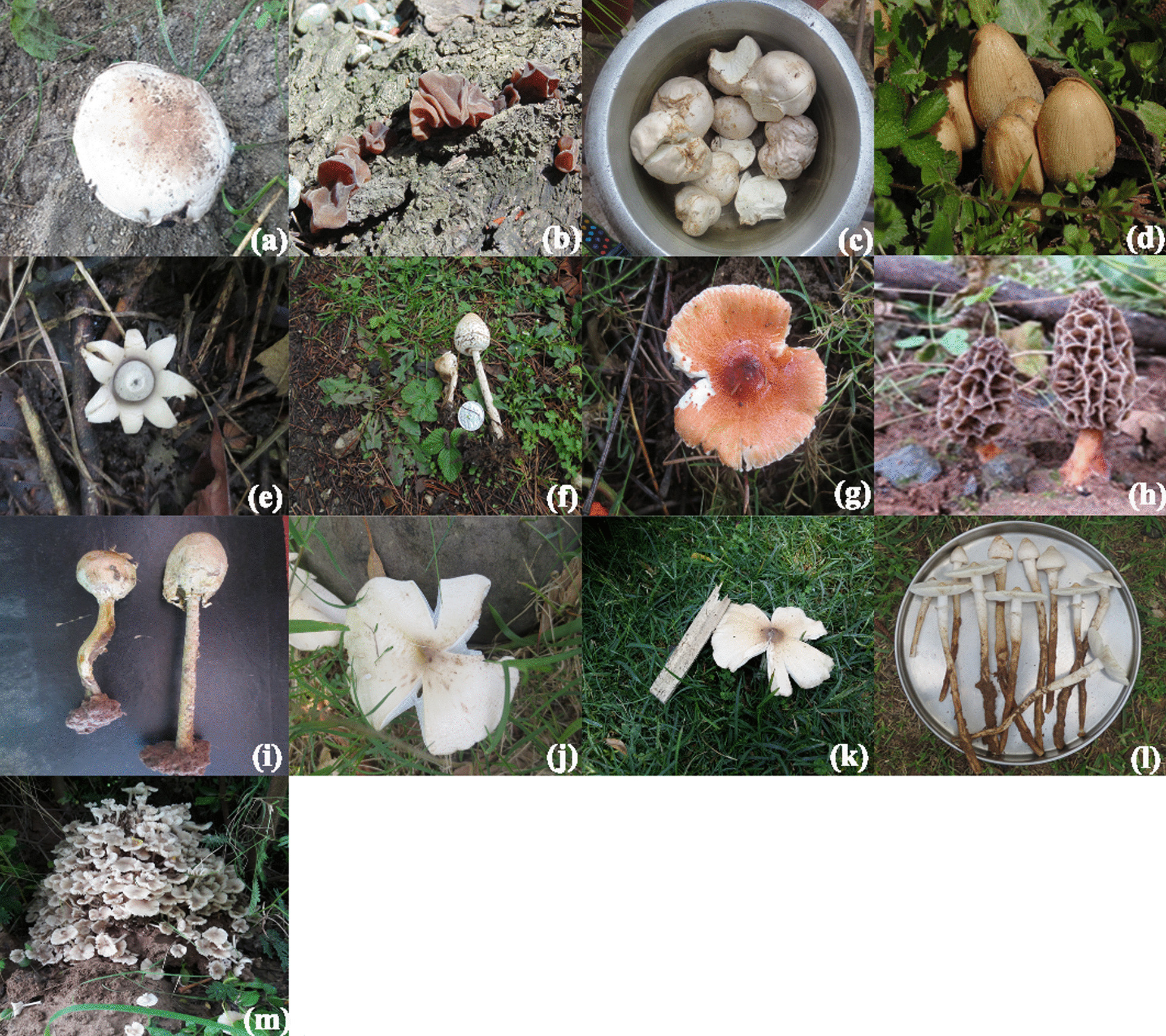
**a**–**m**: **a** *Agaricus californicus* Peck, **b** *Auricularia auricula-judae* (Bull.) Quel, **c** *Calvatia bovista* (L.) Pers., **d** *Coprinellus micaceus* (Bull.) Fr., **e** *Geastrum saccatum* Fr., **f** *Lepiota procera* (Scop.) Gray, **g** *Leucoagaricus rhodocephalus* (Berk.) Pegler, **h** *Morchella esculenta* (L.) Pers., **i** *Podaxis pistillaris* (L.) Fr., **j** *Termitomyces clypeatus* R. Heim, **k** *Termitomyces eurrhizus* (Berk.) R. Heim, **l** *Termitomyces heimii* Natarajan, and **m** *Termitomyces striatus* var. *annulatus* R. Heim

**Fig. 3:**
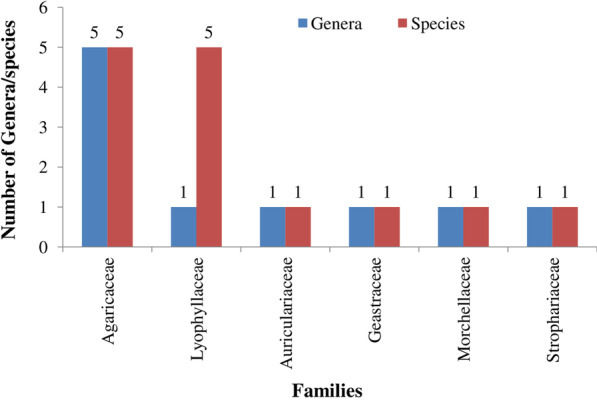
Number of genera and species in various families of fleshy fungi

Total fourteen species (Table 2; Fig. 2a–m) of WEM belonging to six families and ten genera are used by the inhabitants of Jammu. Out of these species, eleven (78.6%) WEM are new records for Jammu and Kashmir. Agaricaceae with 5 genera and 5 species, and Lyophyllaceae with 1 genus and 5 species were the most represented families (Fig. 3). Termitomyces was the largest genera with five species (45.5%).

**Table 2:** Ethnomycology and folk taxonomy of WEM of district Jammu

Scientific name	Family	Vaucher no	Folk name	Fruiting	Uses (no. of informants cited the fungi for a use)	UR
*Agaricus californicus* Peck	Agaricaceae	HBJU405	*Chatri*	Jul–Sep	Culinary (53); medicine (9); gastrointestinal disorders (6)	68
*Auricularia auricula-judae* (Bull.) Quel	Auriculariaceae	HBJU442	*Kankich*	Apr–Jun	Culinary (9); medicine (3)	12
*Calvatia bovista* (L.) Pers	Agaricaceae	HBJU407	*Khucoon*	Apr–Jun	Culinary (31); medicine (11); gastrointestinal disorders (3)	45
*Coprinellus micaceus* (Bull.) Fr	Agaricaceae	HBJU414	*Guchatar*	Jan–Jun	Culinary (13; medicine (3)	16
*Geastrum saccatum* Fr	Geastraceae	HBJU446	*Zameeni Tare*	Jul–Sep	Culinary (19); medicine (6)	25
*Lepiota procera* (Scop.) Gray	Agaricaceae	HBJU415	*Chatri, Khumb*	Jul–Sep	Culinary (13); medicine (6)	19
*Leucoagaricus rhodocephalus* (Berk.) Pegler	Agaricaceae	HBJU418	*Lal Chatri*	Jul–Sep	Culinary (47); medicine (11)	58
*Morchella esculenta* (L.) Pers	Morchellaceae	HBJU404	*Guchii*	Jul–Sep	Culinary (14); medicine (7)	21
*Podaxis pistillaris* (Peck) Hesler	Strophariaceae	HBJU422	*Khumbhi*	Jul–Sep	Culinary (42); medicine (8)	50
*Termitomyces clypeatus* R. Heim	Lyophyllaceae	HBJU427	*Khumb*	Jul–Sep	Culinary (72); medicine (10); gastrointestinal disorders (2)	84
*Termitomyces eurrhizus* (Berk.) R. Heim	Lyophyllaceae	HBJU428	*Khumb*	Jul–Sep	Culinary (49); medicine (11)	60
*Termitomyces heimii* Natarajan	Lyophyllaceae	HBJU429	*Naadu*	Jul–Sep	Culinary (83); medicine (10)	93
*Termitomyces* sp.	Lyophyllaceae	HBJU432	*Tanna*	Jul–Sep	Culinary (86); medicine (24)	110
*Termitomyces striatus* var. *annulatus* R. Heim	Lyophyllaceae	HBJU431	*Sootree*	Jul–Sep	Culinary (65); medicine (10)	75

In the present study, most of the local respondents did not come out with a good deal of descriptive vocabulary with respect to morphology, growth, and habit of macrofungi. As represented in Table 2, there were some local names that were used for a group of fungi, e.g. agarics were commonly known as ‘*Chatri’*, puffballs as ‘*Khucoon’*, and earthstars as ‘*Zameeni Tare*’. Among agarics, *Termitomyces* species were particularly known as ‘*Khumb’*, ‘*Tanna’*, ‘*Sootree’* or ‘*Naadu’*.

**Fig. 4:**
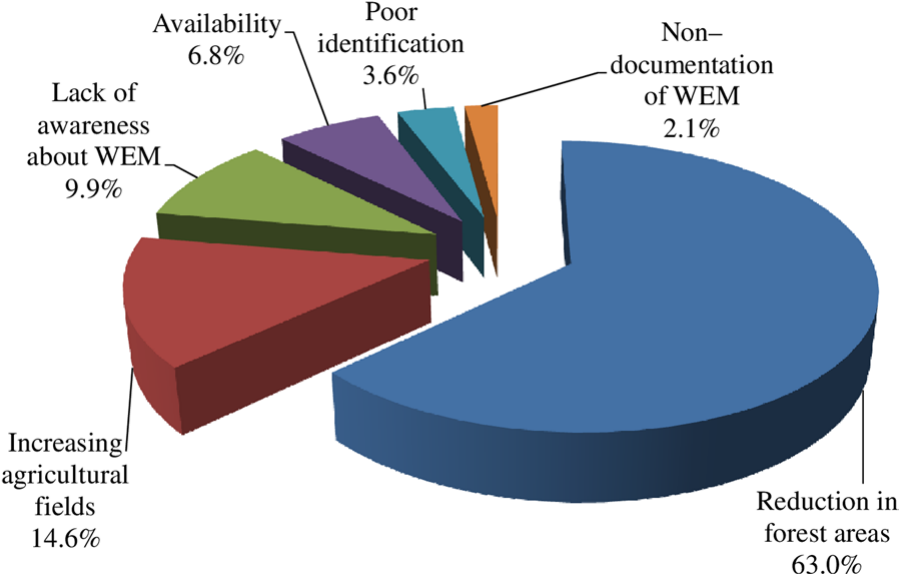
View points of the informants regarding the lesser number of WEM in the study area

As per the informants, reduction in forest areas (63% informants) is the prime reason for the lesser number of WEM in the study area (Fig. 4). Other prominent reasons were increasing agricultural fields (14.6% informants), lack of awareness about the local diversity of WEM among people (9.9% informants), and availability of fungal species in less quantities (6.8% informants).

As per most of the informants (> 73.4%), thundering and lightning are the prime indicators of fruiting of WEM. In the rainy season, they visit the termite mounds, wastelands, grazing lands, and nearby forests, if present, after thundering and lightening (Table 3). Visits after thundering fetched significantly (*P* < 0.05) higher number of WEM in case of males (3.5), elderly (4.5), and illiterate (5.5) informants (Table 2).

**Table 3:** The social belief regarding collection of wild edible mushrooms (WEMs)

Attributes	Informants	Number	Mean number of WEM collected		ANOVA	
			Visits after lightening	Regular visits	*F* value	*P* value
Gender	Female	87	4.6	4.1	1.86	0.177^ ns^
	Male	105	3.5^a^	2.8^b^	5.71	0.019*
Age	Up to 50 years	93	3.3	3.2	0.08	0.781^ ns^
	> 50 years	99	4.5^a^	3.3^b^	5.37	0.023*
Education	Illiterate	61	5.5^a^	4.0^b^	15.99	< 0.001**
	Literate	131	3.3	2.9	2.72	0.101^ ns^

Fisher’s least significant difference (LSD) was applied as multiple-range test when analysis of variance (ANOVA) was found significant at *P* < 0.05

*Ns* non-significant

*^,^**P < 0.05 and 0.001, respectively

The highest CI was recorded for *Termitomyces* sp. (CI 0.57). Other important edible mushrooms were *Termitomyces heimii* (CI 0.48), *Termitomyces clypeatus* (CI 0.44), and *Termitomyces striatus* var. *annulatus* (CI 0.39) (Table 4). All these edible species have some medicinal value. Eleven species each were good against skin problems and development of immunity, and eight species for heart ailments. Some of WEM were reported to have gastrointestinal irritation or mild toxicity.

**Table 4:** Cultural importance index (CI) for WEM of Jammu

Name of the WEM	CI_cul_	CI_med_	CI_gas_	CI_total_
*Agaricus californicus* Peck	0.28	0.05	0.03	0.36
*Auricularia auricula-judae* (Bull.) Quel	0.05	0.02	–	0.06
*Calvatia bovista* (L.) Pers	0.16	0.06	0.02	0.24
*Coprinellus micaceus* (Bull.) Fr	0.07	0.02	–	0.08
*Geastrum saccatum* Fr	0.10	0.03	–	0.13
*Lepiota procera* (Scop.) Gray	0.07	0.03	–	0.10
*Leucoagaricus rhodocephalus* (Berk.) Pegler	0.24	0.06	–	0.30
*Morchella esculenta* (L.) Pers	0.07	0.04	–	0.11
*Podaxis pistillaris* (L.) Fr	0.22	0.04	–	0.26
*Termitomyces clypeatus* R. Heim	0.38	0.05	0.01	0.44
*Termitomyces eurrhizus* (Berk.) R. Heim	0.26	0.06	–	0.31
*Termitomyces heimii* Natarajan	0.43	0.05	–	0.48
*Termitomyces* sp.	0.45	0.13	–	0.57
*Termitomyces striatus* var. *annulatus* R. Heim	0.34	0.05	–	0.39

CI_cul_, CI_med_, and CI_gas_ is cultural importance index of culinary, medicinal, and gastrointestinal disorders, respectively

The maximum consensus (0.98, *F*_ic_), with 596 citations, was recorded for the use of fleshy fungus as culinary purposes (Table 5), whereas the minimum homogeneity was found for immunity development (42 citations and 0.76 *F*_ic_).

**Table 5:** Factor informant consensus (*F*_ic_) of various use categories for fleshy fungi

Use category	*n*_ur_	*n*_t_	*F*_ic_
Culinary	596	14	0.98
Gastrointestinal disease	12	3	0.82
Heart disease	37	8	0.81
Immunity development	49	11	0.79
Skin diseases	42	11	0.76

*n*_ur_ is number of use reports and *n*_t_ is the number of taxa

## Discussion

Agriculture is the main source of livelihood and earning of the inhabitants besides cattle rearing and poultry at small scale. Females help their male counterparts in different agricultural activities like sowing, harvesting and threshing of crops, and storage of grains. They also look after the cattle and along with their children take the livestock to the nearby forests or grazing lands for grazing every morning and generally collect firewood, non-wood forest products and WEM when they return home. The tradition of accompanying children during the collection of non-wood forest products and WEM also transmits vital information about these valuable resources to the next generation. Kumar and Sharma [11] and Bhatia et al. [46] have also reported similar traditions for females and children in other parts of Jammu and Kashmir.

Fourteen WEMs are utilized in the present study. These results are in accordance with Isaan Province of Thailand [47], Yunnan, China [48, 49], Tibet, China [50], Aegadian Islands [51], and Armenia [52]. However, the number of WEM is less as compared to 41 WEM reported from Bhaderwah [11], and 33 [35] and 35 species [34] from Kashmir regions of Union Territory of Jammu and Kashmir. Some other studies worldwide have also recorded higher usage of WEM; 54 species from Lao PDR [53]; 40 [54] and 90 species [55] from Mexico; 56 species from Poznan [56], 32 species from Rzeszow [57], and 65 species from Mazovia [58] regions of Poland; 38 species from Budapest, Hungary [59, 60]; 17 species from Qinling Mountains, China [61]; 22 species from Tibet, China [62]; and 29 species from Sagarmatha National Park, Nepal [63].

*Morchella esculenta* (L.) Pers., and *Termitomyces* spp. are the main species sold in the local market. Collection of WEM is generally at a very small scale and for a limited period in Jammu district. These are collected mainly for household utilization and seldom sold in the local market and therefore are not the significant contributors to the economy of the informants. However, the cultivation of *Agaricus bisporus* (J.E. Lange) Imbach (button mushroom) is common in the district. The same is sold in the market @ Rs. 200 kg^−1^ ($ 2.5 kg^−1^) and is a good source of economy for the locals.

All the fungal species are consumed fresh, due to their perishing nature, except *Morchella esculanta*, which is utilized in both fresh and dried forms. *Morchella esculanta* is generally dried for consumption during harsh cold seasons when the availability of protein rich food is scarce [11].

Agaricaceae and Lyophyllaceae were the most represented families. The higher use of members of Agaricaceae and Lyophyllaceae is in line with other studies [30, 64–66]. Higher percentage of these two families in most of the regional ethnomycology may be to their appealing taste and better income [11, 30] and/or easy to identify as edible, and found on definite locations like termite mounds. However, members of some of the important WEM families like Amanitaceae, Boletaceae, and Russulaceae that are commonly used in other parts of the world [55, 63, 67] were not found at all in Jammu. The reason for this is non-availability of species from these families in Jammu district [68]. Kumar and Sharma [11] have reported members of all these families from Bhaderwah, Pala et al. [34] have recorded only Amanitaceae, and Malik et al. [35] found only Boletaceae from Kashmir Himalayan regions of J&K (UT).

Termitomyces was the largest genus. The dominance of *Termitomyces* spp. is in accordance with most of the studies conducted in the tropical regions [29, 30, 66, 67, 69] due to their flavour, taste, and nutritional values [29].

Female informants accounted for a significantly higher number of WEM in the present study. These findings are in corroboration with studies in Jammu and Kashmir [11, 47], India [15–26], and other parts of the world [29, 30, 49, 70] where female informants had higher knowledge of WEM than males. Garibay-Orijel et al. [71], who reviewed 80 ethnomycological studies, also reported a greater role of women worldwide in mushroom collection, processing, and marketing. However, some studies carried out in Poland [58, 72], China [61], and Africa [73–75] have reported that men are significantly more knowledgeable regarding WEM than women as they can move deep into the forest without any fear [76], whereas few others [77, 78] say that, there is no significant difference between the genders *vis*-*a*-*vis* knowledge of WEM.

Elderly and illiterate informants were also having significantly higher knowledge of WEM than young and literate informants, respectively. A number of other studies in Jammu and Kashmir [11, 46, 79], India [15–26], and other countries [29, 30, 71] have also reported the higher role of elderly and illiterate informants in the collection of non-wood forest products and WEM.

As per the informants, in the present study, reduction in forest areas, increasing agricultural fields, lack of awareness about the local diversity of WEM, availability of fungal species in less quantities, poor identification, and non-documentation of edible and medicinal species of macrofungi have been implicated in mushroom underutilization and some degree of inconsistencies in their usage. Kour [12], Akpaja et al. [27], and Teke et al. [29] have also reported anthropogenic disturbances, reduction in the forest area, and increasing urbanization as the major factors responsible for low diversity of macrofungus in their studies. Giri and Rana [63], however, think that unmanaged harvesting and climate change may be the reasons for decline in the availability of mushrooms in Nepal.

Thundering and lightning are the prime indicators of fruiting of WEM. Most of the elders (88.9%), having more than 50 years of age, believe that these natural phenomena are responsible for bringing up WEM from the lap of mother earth. In the rainy season, they visit the termite mounds, wastelands, grazing lands, and nearby forests, if present, after thundering and lightening. Other workers [29, 30] have also reported thundering and lightning as an important indicator for mushroom hunting.

Another local perception regarding mushroom hunting is that while collecting wild edible fungus one should be silent to ensure that these mushrooms may appear in the next season at the same place. Kumar and Sharma [11] have reported that in the hilly tracks of Doda and Bhadarwah regions of Jammu and Kashmir, the tribes collect the mushrooms, especially morels, early in the morning.

Local people also broadly classified the use of white coloured mushrooms as edible while bright coloured mushrooms are considered poisonous. Some of the elderly informants said that they distinguish the edible fungus by their mild taste. These results are in line with Kumar and Sharma [11], Sagar et al. [16] and Sitotaw et al. [30] who have also reported colour of the mushroom as the prime indicator for identification of WEM.

The knowledge related to the folk nomenclature was scarce and limited in the study area in comparison with the other mycophilic regions of the state like Bhaderwah, Kishtwar, and Ladakh where people had developed rich ethnotaxonomic knowledge and experience in the utilization of the wild edible mushroom resources. Kumar and Sharma [11] have thrown light on 37 vernaculars indigenously used for 71 wild mushrooms from Bhadarwah region of Jammu and Kashmir while as Dorjey [13] reported 45 vernaculars used for various mushroom species in three areas of Ladakh. Kour [12] also recorded different vernacular names like ‘*Zameeni Tare*’ (*Astraeus hygrometricus*), ‘*Santri Chattri*’ (*Leucoagaricus rubrotinctus*), ‘*Sootree’* (*Termitomyces heimii*) from Poonch district.

The highest CI was recorded for *Termitomyces* spp. *Termitomyces* spp. has wide acceptability worldwide due to high concentration of proteins, vitamins, and minerals [67, 80–82], lower fat contents and carbohydrates [83], and an important source of income [84]. All these species grow on or around the termite mounds. As per Hindu religion, these termite mounds are sacred places where '*Naag Devta*' (snake deity) lives and people don’t disturb them and offer water and milk, and *roat* (a traditional chapatti made up of wheat flour, *jaggery*, and *desi ghee*) on every Sunday. Thus, a religious belief provides protection and good nourishment to the fungal mycelium. *Calvatia bovista* (CI 0.24), with a very restricted distribution in the study area, was eaten only in the young stages as some of the people were of the opinion that its consumption in later stages could cause gastrointestinal problems since they were prone to insect infestation when extended fully.

Traditionally, locals follow the *Ayurveda* system for the treatment of diseases. The use of fungi is not reported in local *Ayurvedic* preparations. As per informants, consumption of WEM is good for skin problems, immunity development, and heart ailments due to nutritive values of these fungi. In some other parts of India, *Termitomyces heimii* is used in treatment for cold, fever, and fungal infections [85] and as blood tonic [86], and *Termitomyces eurrhizus* is used for lowering hypertension and curing of rheumatic pains and diarrhea [87]. The fruiting bodies of *Podoxis pistillaria* are used against sunburn and the treatment of inflammation and skin diseases [88], and they also show antibacterial and antifungal activities [89, 90]. Edible and medicinal value of *Calvatia bovista*, *Geastrum saccatum*, *Leucoagaricus rhodocephalus*, and *Morchella esculanta* has also been reported by researchers in other parts of the country and elsewhere in the world [8, 20, 28, 91–95].

Some of WEM, viz. *Agaricus californicus* (CI_gas_, 0.03), *Calvatia bovista* (CI_gas_, 0.02), and *Termitomyces clypeatus* (CI_gas_, 0.01), were reported to have gastrointestinal irritation or mild toxicity. Mild toxicity of these species has also been mentioned by few authors [8, 94, 95], but poisoning is restricted to gastrointestinal upset in a few individuals, a statement well supported by fewer citations in the present study.

The maximum consensus was recorded for the use of fleshy fungus for culinary usage. Similar findings have also been reported by Sitotaw et al. [30] in the community of district Menge of Ethiopia where WEM was utilized primarily for culinary purposes. The minimum homogeneity was found for immunity development (42 citations and 0.76 *F*_ic_). The high values of the factor informant consensus indicate greater homogeneity and also show that informants share whatever knowledge lies with them about WEM [79].

## Conclusion

This is the first-ever study to document the traditional knowledge of wild edible mushrooms (WEM) in district Jammu. Substantial information regarding the usage of wild mushrooms as food and medicine is available with the inhabitants of district Jammu. A total of eleven WEM from 5 families and 7 genera were reported by 50 informants. But there is a great risk of losing this valuable information in near future because females, elders, and illiterate persons were having significantly higher information about WEM than others and all these sects of a society are the most vulnerable as far as storage and spread of information is concerned. In addition to this, reduction in natural habitats and no written record of WEM may also result in erosion of the traditional knowledge about these valuable treasures of nutrition. Although accompanying children with mothers is a small ray of hope for maintaining perpetuity of knowledge regarding WEM, still we need to safeguard the natural habitats of mushrooms and popularize them as early as possible. As reported in other tropical regions of the world, *Termitomyces* was the most dominant genera of the present study. For the betterment of the society and to fulfill the requirements of both income generation and food security, we need to focus our research on the domestication and cultivation of *Termitomyces* spp. A detailed investigation with respect to nutritional as well as medicinal aspects of these species is also required.

## Data Availability

All data generated or analysed during the conduct and writing up of the manuscript are incorporated in the research article.

## Abbreviations

ANOVA: Analysis of variance
CI: Cultural importance index
FAO: Food and Agriculture Organisation of United Nations
F_ic_: Factor informant consensus
J&K: Jammu and Kashmir
KOH: Potassium hydroxide
LSD: Least significant difference
UR: Use reports
UT: Union Territory
WEM: Wild edible mushrooms
